# Evaluating Microclimate Modification and Acute Cardiovascular Stress Responses to a Dense Urban Microforest: The Green Oasis (GRO) Protocol

**DOI:** 10.3390/ijerph23030365

**Published:** 2026-03-13

**Authors:** Rachel Keith, Sean Willis, Natalie Christian, Farzaneh Khayat, Jackie Gallagher, William Scott Gunter, Julia Kachanova, Andrew Mehring, Rachel Pigg, Doris Proctor, Allison E. Smith, Cameron K. Stopforth, Patrick Piuma, Ted Smith, Aruni Bhatnagar

**Affiliations:** 1 Christina Lee Brown Envirome Institute, University of Louisville, 302 E Muhammad Ali Boulevard, Louisville, KY 40202, USA; rachel.keith@louisville.edu (R.K.); sean.willis@louisville.edu (S.W.); farzaneh.khayat@louisville.edu (F.K.); jacqueline.gallagher@louisville.edu (J.G.); allison.metz@louisville.edu (A.E.S.); cameron.stopforth@louisville.edu (C.K.S.); patrick.piuma@louisville.edu (P.P.); ted.smith@louisville.edu (T.S.); 2 Division of Environmental Medicine, Department of Medicine, University of Louisville, Louisville, KY 40202, USA; 3 Urban Design Studio, University of Louisville, Louisville, KY 40202, USA; 4 Department of Biology, University of Louisville, Louisville, KY 40202, USA; natalie.christian@louisville.edu (N.C.); julia.kachanova@louisville.edu (J.K.); andrew.mehring@louisville.edu (A.M.); rachel.pigg@louisville.edu (R.P.); doris.proctor@louisville.edu (D.P.); 5 Department of Geographic and Environmental Sciences, University of Louisville, Louisville, KY 40202, USA; william.gunter@louisville.edu; 6 Center for Healthy Air Water and Soil, University of Louisville, Louisville, KY 40202, USA

**Keywords:** microforest, cardiovascular, urban heat, climate, heart rate variability, stress

## Abstract

**Highlights:**

**Public health relevance—How does this work relate to a public health issue?**

- Green space exposure provides extensive benefits for physiological and mental health that will become more important as global temperatures rise and populations continue to urbanize.
- Small-scale, targeted greening interventions and their influence on temperature-induced cardiovascular stress are not well understood.

**Public health significance—Why is this work of significance to public health?**

- This protocol focuses on the development of a comprehensive framework to assess the impact of targeted greening on urban microclimates, ecological function, and cardiovascular resilience.
- Highlights the work needed to understand how small green spaces can impact users and the surrounding urban form.

**Public health implications—What are the key implications for practitioners, policy makers, and/or researchers?**

- Final results will provide insight into the effectiveness of greening interventions on small, underutilized urban lots, which are common in American downtowns.
- Policymakers can utilize this framework to analyze outcomes related to different green spaces in similar urban environments.

**Abstract:**

The Green Oasis (GRO) Project is a targeted urban greening intervention designed to evaluate the environmental and health impacts of compact, high-density plantings in dense built environments. Initiated in downtown Louisville, the project transformed Founders Square, a 0.64-acre sparsely planted park, into a microforest (“Trager Microforest”), a multilayered planting of 119 trees and more than 200 shrubs. The impact of this intervention is being assessed through a randomized crossover study in which participants walk in the microforest and a nearby impervious parking lot. Physiological outcomes include heart rate, heart rate variability, arterial stiffness, and stress biomarkers measured in saliva, urine, and sweat. Environmental conditions are continuously monitored by fixed and mobile weather stations, air pollution sensors, and biodiversity surveys. Baseline assessments were conducted in 2023 and 2024, with post-planting evaluations now underway (2025–). Power calculations indicate adequate sensitivity (*n* ≈ 40–50) to detect changes in cardiovascular stress responses in participants. Complementary ecological measurements include soil microbiome composition, greenhouse gas fluxes, and avian diversity. This study addresses critical gaps in understanding how small-scale, high-density greening interventions affect cardiovascular resilience, stress physiology, and microclimatic regulation. By integrating environmental, biological, and human health data, GRO establishes a comprehensive framework for evaluating the efficacy of urban microforests as nature-based solutions. The results are expected to inform urban planning, public health strategies, and climate adaptation policies, demonstrating how compact greening interventions can simultaneously mitigate heat, reduce pollution, enhance biodiversity, and promote human wellbeing in dense urban cores.

## 1. Introduction

Extensive evidence indicates that exposure to greenness benefits human health [1]. Individuals living in greener areas show lower levels of stress [2,3,4], depression [5,6,7], insulin resistance [8], diabetes [9,10,11], stroke [12,13], cancer [14,15,16], cardiovascular mortality [17,18,19], and all-cause mortality [19,20], as well as greater longevity [21]. Higher neighborhood greenness has also been associated with better cognitive function, greater physical activity, and reduced obesity risk [22]. Time spent in natural environments is thought to reduce mental fatigue and enhance emotional wellbeing [23,24], underscoring the broad physical and psychological benefits of contact with nature.

Urban greenery is also critical for climate adaptation. Nature-based solutions, including targeted, evidence-based greening interventions, can buffer adverse climatic impacts on health [25,26,27,28,29,30]. Vegetation cools the urban environment through transpiration, whereby water absorbed by the roots evaporates from leaves, lowering ambient temperatures [31,32,33,34]. A study of 93 European cities estimated that increasing tree coverage to 30% could reduce average summer temperature by 0.4 °C and prevent up to 84% of heat-related deaths [35]. In addition to mitigating heat, vegetation also improves air quality, although the mechanisms vary by species, vegetation health, and pollutant type. Modeling and field studies suggest that trees and shrubs can remove ozone [36], polycyclic aromatic hydrocarbons (PAHs) [37], perfluoro carboxylic acids (PFCA) [38], and NO_2_ [39,40,41,42,43,44], and city-wide estimates indicate that green infrastructure could reduce NOx by 35%, PM_2.5_ by 8%, and PM_10_ by 21% [45]. Trees also function as phytoextractors, removing metals such as Cu [46] and capturing metals such as Cd and Pb from the air [47]. Additional ecosystem services include improving soil quality, supporting biodiversity, and reducing stormwater runoff and flooding. Collectively, these benefits highlight the capacity of greenness to mitigate pollution, enhance climate resilience, and promote health, particularly in populated urban areas.

The urgency of urban greening is underscored by climate projections: by 2100, nearly 74% of the world’s population is expected to experience climatic conditions exceeding health-relevant thresholds for at least 20 days annually [48]. Even modest increases in temperature can have widespread consequences [48]. Acute heat exposure induces hemodynamic responses, such as sweating and cutaneous vasodilation, that could compromise cardiovascular function. In addition to temperature, fluctuations in atmospheric pressure and relative humidity are also linked to cardiovascular events [49,50]. Greening strategies may buffer these risks. Controlled studies of walking in forests [51], natural trails [52], and urban green environments [53,54] have demonstrated cardiovascular benefits such as improvements in arterial stiffness [55], reduced sympathetic nervous activity, increased parasympathetic nervous activity, lower heart rate [53], reductions in systolic and diastolic blood pressure [53], as well as lower urinary noradrenaline and dopamine levels, and increases in serum adiponectin [52].

Despite this growing body of evidence, the combined effects of greenness and temperature on cardiovascular health remain poorly understood [56]. Although seasonal variations in the physiological impacts of walking in urban parks have been reported [57], the extent to which urban green spaces mitigate temperature-related cardiovascular stress is unclear. Moreover, the physiological and environmental impacts of small urban plantings have not been studied before. This gap in understanding is critical, as clusters of vegetation may play an especially important role in improving urban environments and buffering heat in heavily built areas. It remains unknown, however, whether small-scale, high-density greening interventions can meaningfully improve microclimates and cardiovascular function.

To address these gaps, we initiated the Green Oasis (GRO) project, designed to evaluate the environmental and health effects of dense urban plantings such as microforests, groves, or woodlets. Primarily, we will evaluate whether dense urban microforest installation modifies heat exposure and acute cardiovascular stress responses. Using a randomized crossover design, we assess the acute cardiovascular effects of walking in an urban setting before and after the installation of a compact, multilayered microforest. We hypothesize that clustered plantings will attenuate urban heat and reduce cardiovascular stress during exposure. Findings from this study are expected to provide novel insights into how small-scale, high-density greening interventions can improve cardiovascular resilience, helping to foster wellbeing in urban populations.

The primary objective of the Green Oasis (GRO) Project is to determine whether, in comparison with exposure to an impervious parking lot, exposure to an urban microforest improves cardiovascular and stress responses (heart rate variability and blood pressure). The secondary objective is to evaluate how exposure to the microforest affects stress biomarkers (catecholamines and cortisol), rumination, anxiety, and exposure to anthropogenic volatile organic compounds (VOC). Our exploratory analyses are to evaluate the effects of the microforest on surrounding environmental conditions (temperature, humidity, particulate matter, NO_2_, black carbon, and noise) and enhance ecological services (avian density, microbiome, etc). Lastly, the integrated objective is to integrate meteorological, ecological, and clinical data to evaluate whether environmental modifications mediate physiological responses. A summary of all research objectives/aims is listed in Table 1.

## 2. Materials and Methods

### 2.1. Study Site and Context

**Figure 1:**
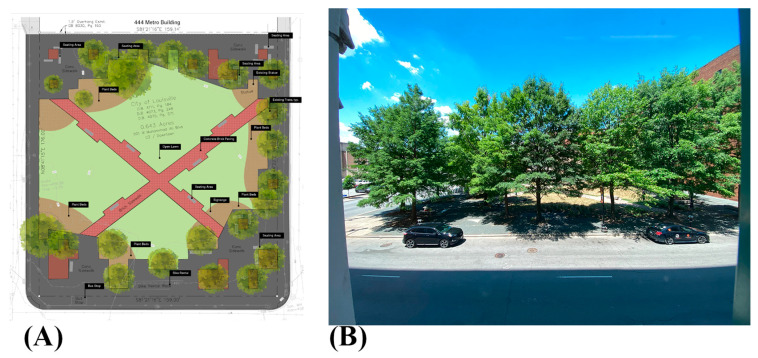
(**A**) Site map of the Founder’s Square located between W Muhammad Ali Blvd and 444 Metro Building; and (**B**) photograph of the Founder’s Square taken from 5th Street, taken July 2022.

The study site is Founders Square in downtown Louisville. In 1960, a two-story circular building housing an information center and a symbolic “Stonehenge” dedicated to the city’s founders was erected at the site. The building has since been demolished, leaving a one-block parcel that was largely underutilized and dominated by 24 mature oak and cherry trees with scattered shrubs. The central space consisted primarily of grass and brick pavers (Figure 1).

Although previously underused, Founders Square offers an ideal setting for evaluating the environmental and health impacts of greenness. The park lies in one of the densest parts of Louisville, adjacent to the city’s largest residential complexes and served by the second-highest ridership bus line. Across the street, extensive asphalt parking lots provide a clear comparison to the microforest, especially given that downtown Louisville is approximately 54% impervious surface. An estimated 2900 residents live within a half-mile radius of the park, and roughly 31,700 people work within that radius on weekdays. The site is owned by the Louisville Metro Government and has been leased to the University of Louisville for a 30-year period, enabling long-term study of the intervention.

### 2.2. Community Engagement

Community input was central to the design of the microforest. A two-day design workshop and charrette invited nearby residents, downtown workers, and local professionals to collaborate with the contracted design team from Gresham Smith, a landscape architecture, planning, engineering, and architecture firm. The workshop consisted of seven sessions containing roundtable discussions, guest speakers, site visits, and an open house. Researchers working on the Trager Microforest were in attendance to interact with participants and provide feedback to the design firm. Specific themes such as health promotion, heat mitigation, public access, and social connection were discussed. Gresham Smith then compiled results from the workshop/charrette into a summary document, which then informed the final master plan for the space.

The summary document containing feedback from approximately 40 participants informed the initial designs and helped establish key design priorities. Many elements ultimately implemented were first conceived during this workshop, such as the use of Chronolog picture stations, informational signage, and furniture layout/design. General priorities of participants also informed the design, with concepts such as health promotion, natural immersion, and social connection being brought up often. These discussions informed design elements, such as seating placement, layered planting density, public access, signage, and pathway configuration.

To promote transparency and community involvement, dedicated webpages were created to share information about the project. The forest was named the “Trager Microforest” in recognition of the main supporting donor’s family. Webpages were made accessible through QR codes posted on construction signage around the site, and weekly updates were published on the University of Louisville Urban Design Studio website, providing progress reports on both scientific research and construction activities.

### 2.3. Environmental Data Collection

#### 2.3.1. Meteorological Monitoring

To capture meteorological conditions, we deployed two types of weather stations: a WiFi-based “prosumer” system (Tempest Weather Station, WeatherFlow Inc., Santa Cruz, CA, USA) and a research-grade system (Campbell Scientific Inc., Logan, UT, USA). The Tempest stations record a wide range of variables (Table 2) at 1 min intervals and automatically transmit data to a hub and redundantly log in cloud storage. In summer 2023, six Tempest stations were installed, four at the corners of the site and two near the center, allowing measurements across varied shade and ground-cover conditions.

To ensure accuracy and provide long-term monitoring, a Campbell Scientific research-grade station was installed at the site’s center. This station records many of the same meteorological variables as the Tempest, but its temperature sensor is housed in a fan-aspirated shield to prevent stagnation bias. Data are recorded at 1 min intervals and manually downloaded from the datalogger. A fine temporal resolution is used, corresponding to participant exposure periods as part of the human crossover component. Site-wide microclimatic analysis will use hourly and daily data, which can be aggregated into monthly datasets for long-term analysis. Both the Tempest and Campbell systems collected baseline data until site construction began. The central research station and two Tempest units were removed for construction; the research station was reinstalled in December 2024, while the central Tempest stations were permanently eliminated from the final design (Supplemental Figure S1).

During the second round of clinical trials, before the Campbell station was reestablished, a mobile cart system was developed to replicate the fixed-station measurements. This was done to ensure continuity of meteorological measurements when the central fixed station had to be removed during construction. The mobile unit collected equivalent variables at 1 min intervals and was activated at least 30 min before participant arrival to ensure stabilization. In parallel, one researcher wore an AirBeam3 device during participant walks, providing second-by-second measurements of particulate matter (PM_1_, PM_2.5_, and PM_10_).

Both the fixed and mobile Campbell stations were calibrated following manufacturer guidelines. Parallel deployment of the Campbell and Tempest stations allowed for cross-validation during baseline measurements. Data will be acquired both at the microforest site and the impervious parking lot to obtain concurrent measurements. All data will go through quality control screening for missing values, sensor drifts, sensor faults, and anomalous spikes.

#### 2.3.2. Spatial Characterization of Vegetation Structure (LiDAR)

**Figure 2:**
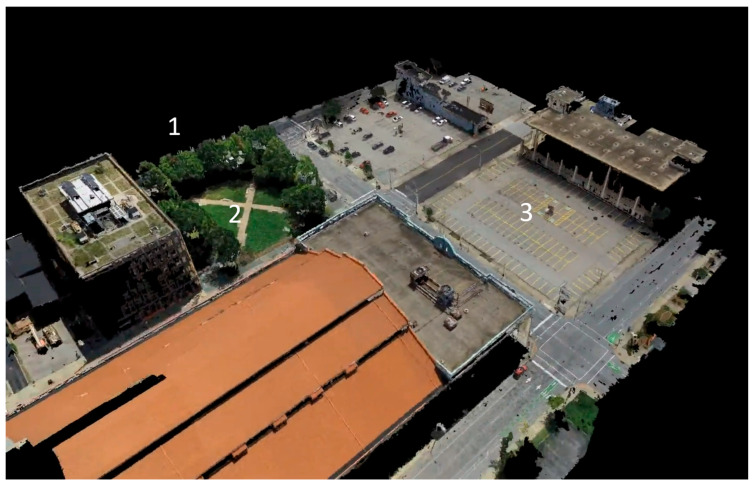
LiDAR scan of the Founder Square prior to installation of the Trager Microforest (May 2023). (1) Meeting site for clinical study participants (not in point cloud). (2) Founders Square. (3) The “urban” space used as comparison in the clinical study.

LiDAR scans have been and will continue to be acquired periodically as the microforest gets established. An initial LiDAR scan was performed in August 2022 utilizing a drone (Figure 2). This was followed by a scan in January 2023, prior to construction, and another in May 2024 during construction. The last two scans utilized a backpack LiDAR device to more effectively capture vegetation density, which will also be used for future scans. The LiDAR data will allow for the structural characterization of vegetation density, canopy development, and quantification of three-dimensional vegetation before and after intervention.

### 2.4. Biological Data Collection

#### 2.4.1. Avian Surveys

To establish baseline biodiversity and enable longitudinal comparison, avian surveys were conducted prior to intervention. For comparison, data were also collected from two other Louisville green spaces: the University of Louisville Humanities Quad and Nettleroth Bird Sanctuary in Cherokee Park. From February through April 2024, each site was visited >15 times (morning and late afternoon/early evening), with 30 min observation periods per visit. Birds were identified to species visually using high-quality binoculars (Swarovski CL Companion 8 × 30, Vortex Razor UHD 10 × 42, Eagle Optics Ranger ED 10 × 42, and Nikon 10 × 42). The abundance of each species was also estimated.

#### 2.4.2. Soil Microbiome Data and Greenhouse Gas Fluxes

Soil microbial activity and gas fluxes were assessed using a Los Gatos Research Ultraportable GHG Analyzer to measure CO_2_, CH_4_, and water vapor concentrations (the latter for correction). Flux chambers (~5.7 L and ~4.6 L volumes) connected to soil collars (27 and 22 cm diameter) allowed solar penetration and captured plant–soil gas exchange. Net emissions were inferred from increasing chamber concentrations, while decreasing levels indicated uptake. To minimize disturbance, collars were set 30 min before measurements. Sampling occurred on sunny days between 10:00 and 18:00 to capture peak photosynthetic activity. Photosynthetically active radiation (PAR) was measured using a Li-Cor LI-190R sensor (LICORbio Inc., Lincoln, NE, USA). After each light-phase measurement, the chamber was covered to create dark conditions (PAR < 1 μmol m^−2^ s^−1^), enabling separation of soil respiration from photosynthetic uptake. Incubations of 60–90 s were sufficient to capture flux changes while minimizing chamber heating and CO_2_ depletion. Soil moisture and temperature were recorded using a Stevens Hydra-Probe II (Stevens Water Monitoring Systems, Inc., Portland, OR, USA), and air temperature inside chambers with an Onset HOBO logger (Onset Computer Corporation, Bourne, MA, USA).

#### 2.4.3. Soil Sampling and Microbiome Characterization

We are also assessing microbial (bacterial and fungal) community composition and diversity in the microforest over time. This is being done to characterize microbial community health and composition pre- and post-intervention as an exploratory outcome related to ecosystem resilience. Samples were and will be repeatedly taken from the site over time from the same locations; these sample sites cover each corner of the site, as well as the center. From each collected soil sample, we take a 0.25 g subsample of soil and extract genomic DNA using the Qiagen DNeasy^®^ PowerSoil^®^ Kit (Qiagen N.V., Hilden, Germany) following the manufacturer’s guidelines. DNA will be shipped to the W. M. Keck Center for Comparative and Functional Genomics at the University of Illinois, Urbana-Champaign, for Fluidigm amplification, followed by Illumina NovaSeq 6000 sequencing. For bacteria, we will amplify the 16S rRNA region using primer pair V4_515F_806R_New (V4_515F_New 5′-GTGYCAGCMGCCGCGGTAA, V4_806R_New 5′-GGACTACNVGGGTWTCTAAT). For fungi, we will amplify the internal transcribed spacer (ITS) region using primer pairs for ITS1 (ITS1F_ITS2, ITS1F 5′-CTTGGTCATTTAGAGGAAGTAA, ITS2 5′-GCTGCGTTCTTCATCGATGC), and ITS2 (ITS3_ITS4, 5′-GCATCGATGAAGAACGCAGC, ITS4 5′-TCCTCCGCTTATTGATATGC).

#### 2.4.4. Tree Inventory and Ecosystem Services

To quantify ecosystem services, all trees at the site were inventoried. Species were identified, and diameter-at-breast-height (DBH) and crown health (dieback %) were recorded. The U.S. Forest Service i-Tree Eco software (v6.1.47) was used to estimate annual carbon storage and sequestration, pollution removal, oxygen production, and prevented runoff. Carbon storage represents the stock released upon tree death and decomposition, while sequestration reflects annual CO_2_ uptake. Pollution removal estimates were based on species, size, and canopy characteristics. Oxygen production was derived from sequestration data, and prevented runoff was estimated from canopy cover, species type, and crown conditions.

### 2.5. Human Study Design and Participants

#### 2.5.1. Study Design and Timeline

The GRO Project is a randomized crossover study of healthy adults aged 18–75 years. Baseline data were collected in two clinical waves prior to installation of the microforest: Wave 1 (Summer 2023) and Wave 2 (Summer 2024). To evaluate the intervention, a follow-up study is underway in Summer 2025 (Wave 3), with additional longitudinal assessments planned for Summers 2026 and 2027 (Waves 4 and 5).

#### 2.5.2. Participant Recruitment and Eligibility Criteria

Participants were recruited for The GRO Project through online platforms (Twitter, Facebook, and Craigslist) and by posting flyers in downtown Louisville. For follow-up visits, all returners were contacted through phone and email information. Participants were excluded for (i) tobacco use within the last 60 days, (ii) uncontrolled cardiovascular disease (CVD) or CVD risk factors such as hypertension, hyperlipidemia, and diabetes mellitus, (iii) ≥stage 3 renal disease, (iv) active cancer, (v) active illicit drug use, (vi) pregnancy, (vii) major trauma within the past 6 months, or (viii) part of a vulnerable population (i.e., children, prisoner, etc.). Additionally, participants were required to be (i) between the ages of 18–75, (ii) able to spend 1 h outdoors in conditions up to a 104 heat index, (iii) capable of walking one mile without limitations, and (iv) have a body mass index lower than 35. Participants were compensated $50 for completion of each in-person exam. The study protocol was approved by the University of Louisville Institutional Review Board (IRB# 23.0437), and written informed consent was obtained prior to testing.

### 2.6. Study Visit Protocol

#### 2.6.1. Study Characteristics

**Figure 3:**
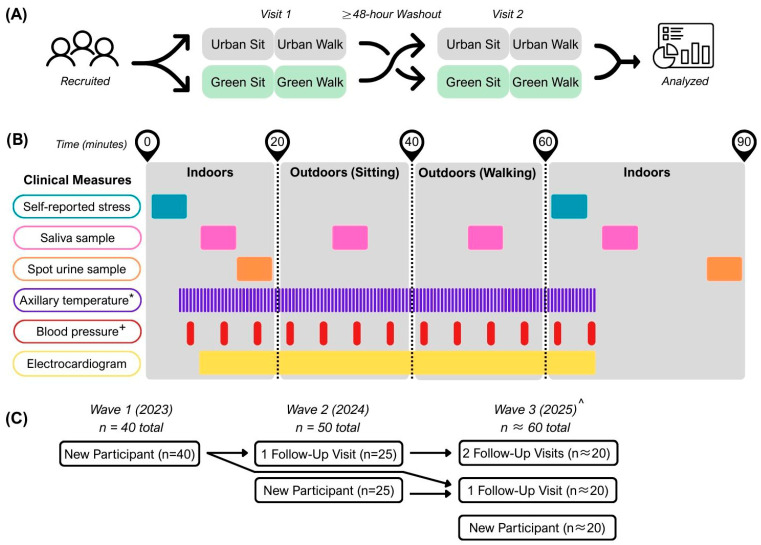
Schematic of clinical study aspects. (**A**) Cross-over study design. (**B**) Protocol for the collection of clinical measures. (**C**) Participants recruited by wave. * Axillary temperature only recorded in Waves 2 and 3. + Different 1. versus Waves 2 and 3. ^ Wave 3 currently in collection; sample size represents anticipated collection.

In Wave 1, we recruited 40 participants, of whom 25 returned for Wave 2. We further supplemented Wave 2 with 25 new participants (Figure 3C). We anticipate collecting another ~60 participants in Wave 3. Demographic characteristics of participants in Waves 1 and 2 are presented in Table 3. Participants from Waves 1 and 2 will be contacted for post-intervention follow-up visits during future waves.

#### 2.6.2. Randomization and Visit Procedures

Trained personnel conducted all study visits. At baseline, we randomized participants to one of two groups, which determined the sequence of conditions: either the microforest (test site), followed by a nearby parking lot (control site), or vice versa (Figure 3A). Testing lasted 40 min for each location, conducted either at 8:30 AM or 10:15 AM to minimize circadian variation. The second visit was temporally matched with the start time of the first visit. Each visit began with electronic questionnaires, application of wearable biomonitors, and baseline collection of biomonitor data and biospecimen sampling. Following baseline measures, we guided participants outside to spend 20 min resting in a chair and 20 min walking at a self-selected leisurely pace. Following the 40 min session, participants returned indoors to complete post-exposure questionnaires and provide additional urine and saliva samples (Figure 3B). The study visits were temporarily separated and conducted on distinct days. Acute thermal and cardiovascular responses (e.g., HRV, blood pressure, and cortisol) are expected to return to baseline within hours following exposure.

#### 2.6.3. Questionnaires and Psychosocial Measures

We administered electronic questionnaires prior to the first visit that included demographic characteristics, medical history, perceived stress, perceptions of nature, and behavioral habits (Supplementary Table S1). On the day of the in-person exam, participants were instructed to refrain from eating or drinking 30 min prior to the start of each visit. Height and weight were measured upon arrival. Each visit began with the State-Trait Anxiety Inventory (STAI) [58] to assess current anxiety levels. For 2024, the Subjective Units of Distress Scale (SUDS) was added to measure distress. For 2025, the Brief State Rumination Inventory (BSRI) [59] was also added to assess rumination.

#### 2.6.4. Physiological and Cardiovascular Measurements

Following questionnaires, we began physiological monitoring, which included ambulatory blood pressure, heart rate (HR), heart rate variability (HRV), axillary temperature, and biospecimen measures. In Wave 1 (2023), blood pressure was recorded every 5 min using a Mobil-O-Graph device (IEM GmbH; Aachen, Germany); in Wave 2 (2024), we switched to an iHealth Neo ambulatory cuff (Vivalink; Campbell, CA, USA) to improve data quality and link backend data temporally under the same Vivalink device network. Both blood pressure devices are FDA-cleared and validated for ambulatory blood pressure measurement, and calibration checks were performed prior to deployment. For Waves 1 and 2, we measured HR and HRV continuously with the multi-vital electrocardiogram (ECG) patch (Vivalink; Campbell, CA, USA) at 128 Hz. We also added axillary temperature in Wave 2, measured by an FDA-approved, clinical-grade patch that records in 12 s intervals (Vivalink; Campbell, CA, USA).

#### 2.6.5. Biospecimen Collection

Spot urine samples were collected to assess chemical exposure and salivary samples to measure cortisol. Spot urine samples were collected at baseline and 30 min post-exposure to allow for metabolism of biotic and abiotic chemicals. Saliva samples (~1 mL) were taken at four timepoints in 25 min intervals, including baseline, seated outdoors, walking outdoors, and post-exposure, to establish a cortisol curve. A detailed summary of all exam procedures is provided in Table 4. Throughout the visits, participants carried an AirBeam personal air quality monitor (HabitatMap), which continuously measured particulate matter (PM_1_, PM_2.5_, and PM_10_), humidity, and temperature. Additional environmental monitoring included a micro-aethalometer for black carbon (BC), a portable gas monitor for NO_2_, a noise monitor, and a HOBO data logger with GPS to track ambient temperature and relative humidity.

### 2.7. Integrated Ecological, Environmental, and Human Health Conceptual Framework

**Figure 4:**
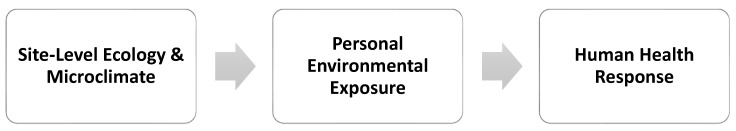
Diagram showing the integrated framework linking the different study approaches for GRO.

This urban microforest intervention modifies local environmental conditions, which changes individuals’ exposure to heat and pollutants during walking. These exposure differences are hypothesized to influence human health (cardiovascular and stress-related) responses measured in the randomized crossover study. As shown in Figure 4, the integrated framework links site-level ecology and microclimate to personal environmental exposure and, ultimately, to human health response.

### 2.8. Greening Intervention: The Trager Microforest

#### 2.8.1. Site Preparation and Infrastructure

**Figure 5:**
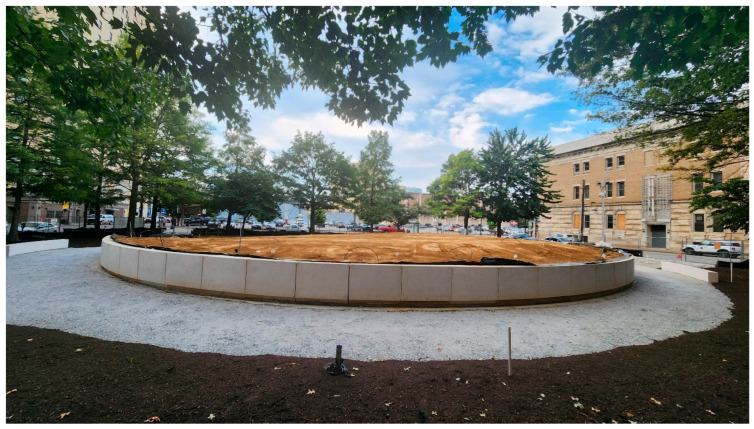
Photograph of the completed retaining wall with soil backfilled in (August 2024).

The intervention began after completion of the first round of baseline testing in Summer 2023. Initial site modifications included the removal of the existing central walkway and the installation of a semi-circular retaining wall, along with a new perimeter walking path (Figure 5). The retaining wall is three feet tall and spans 188 feet, and the wall’s thickness varies from one to two feet to support the weight of the new soil. Benches and lighting were added, and the retaining wall was backfilled with approximately 700 tons of Nugent soil to create a three-foot raised area sloping southward. In addition, metal signage and fins were installed on the south side of the retaining wall to complete the circle, with the signage being 66 inches in height and four inches thick, and the metal fins being 60 inches in height and one inch thick (Supplemental Figure S2). This middle well zone was planted with grass, and mulch was applied along the park’s edges prior to the wave 2 physiological assessment in Summer 2024.

#### 2.8.2. Planting Design and Species Composition:

The primary phase of greening occurred in Winter 2024 and extended into the following months. A total of 119 trees representing 31 species were planted (Supplementary Table S2), comprising a mix of deciduous (primarily Anacardiaceae, Fabaceae, and Fagaceae families) and evergreen species (mainly Pinaceae). This combination was selected to maximize cooling benefits in a temperate climate and enhance year-round canopy cover. In addition, 242 shrubs and hundreds of herbaceous groundcover plants were installed across the site (Supplementary Table S3). Plant material was sourced from nurseries in multiple states and transported directly to Louisville to minimize storage stress prior to planting.

**Figure 6:**
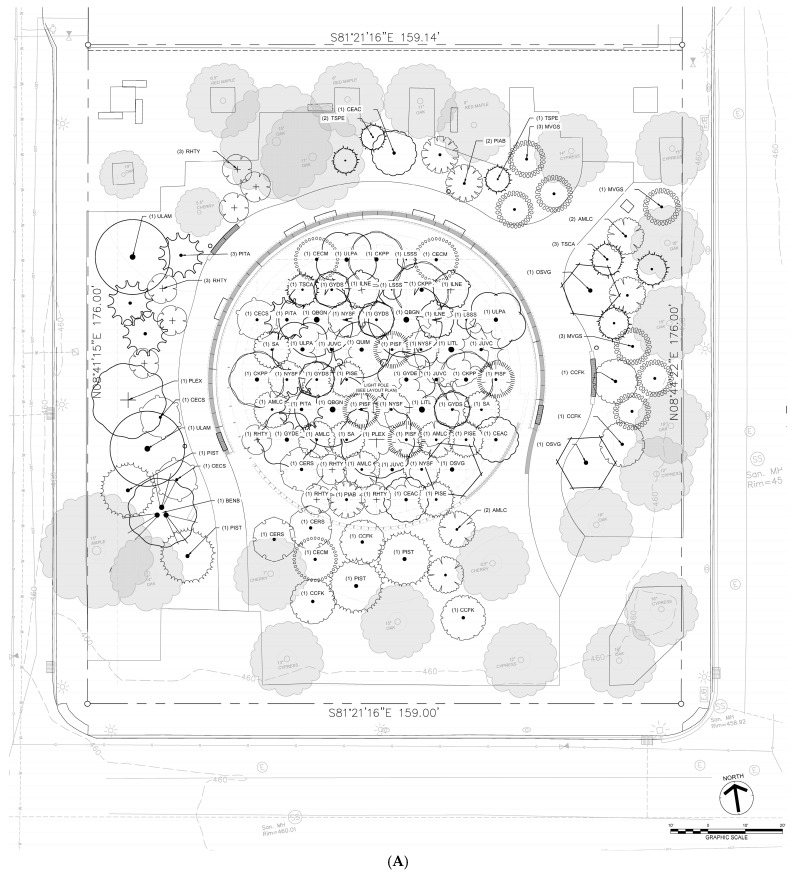

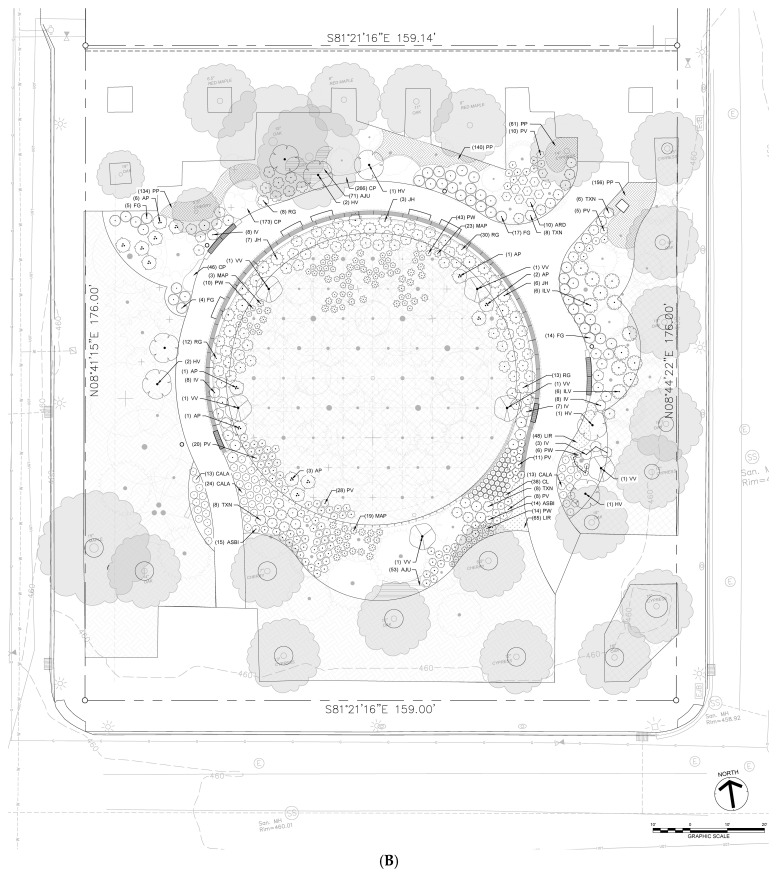
Schematic of the microforest planting design. Panels depict a high-density, multilayered planting arrangement of (**A**) trees and (**B**) shrubs modeled after natural forest succession.

Trees were planted in close spacing (1–1.5 m apart) to accelerate canopy closure. Shrubs were positioned between rows to form a layered understory, while herbaceous species and groundcovers filled interstitial spaces. This stratified planting scheme was designed to mimic natural forest succession, increase soil infiltration, suppress weeds, and promote early biodiversity. Collectively, these dense plantings are expected to enhance evapotranspirative cooling, mitigate urban heat, and improve ecosystem resilience (Figure 6A,B). Progress of installation is shown in Figure S3.

### 2.9. Data Analysis

#### 2.9.1. Statistical Analysis Overview

Descriptive statistics will be calculated for all outcomes, demographic variables, and environmental exposures. Continuous variables will be assessed for normality; skewed outcomes will be transformed as appropriate. Primary analyses will compare personal exposure to temperature, humidity, and particulate matter (PM) between the two walking sites (microforest vs. parking lot) using both personal monitors and fixed/mobile monitoring data.

#### 2.9.2. Primary Analyses

The primary outcomes are changes in HRV and blood pressure. Linear mixed-effects models will be used to evaluate associations between temperature and these outcomes, accounting for the crossover, repeated-measures design. Participants will be modeled as random effects, with treatment (microforest vs. parking lot) and sequence (site order) as fixed effects. An interaction term between temperature and treatment will test whether walking in the microforest modifies temperature–health associations. Linearity of associations will be assessed using splines. Given the change in blood pressure-measuring devices between Waves 1 and 2, the study wave will be included as a covariate in statistical modeling to account for any systemic device-related differences, and sensitivity analyses will assess whether results differ by device. Carryover effects due to insufficient washout in the carryover design will also be evaluated by including sequence and period effects in the mixed-effects models.

Potential confounders will be identified using directed acyclic graphs (DAGs). Models will adjust for age, sex, race, BMI, education, particulate matter levels, and hypertension status. Effect modification by sex and PM concentrations (PM_1_, PM_2.5_, and PM_10_) will also be tested. Sensitivity analyses will examine the influence of medication use and prior smoking history.

#### 2.9.3. Correlation Structure

A covariance structure will be incorporated to account for within-subject correlation across repeated measures during each study day. Changes in health endpoints will be expressed as percent change with 95% confidence intervals.

#### 2.9.4. Secondary Analyses

Direct associations of temperature, humidity, and pollutants with health endpoints during walking will be evaluated using additional mixed-effects models. Exposure metrics will be averaged over defined time intervals, and endpoint changes will be estimated as percent differences.

#### 2.9.5. Exploratory Neighborhood-Level Analyses

Time series data from site monitors and regional models will be used to assess the broader impact of the microforest on local climatic conditions. Two reference groups will be used: (1) Founders Square pre-intervention and (2) the adjacent parking lot. Interrupted time series regression will estimate intervention effects, with the planting date as the interruption point. Weekly aggregated data will be analyzed, and Fourier terms will capture seasonal variation. Future analyses may also explore changes in human mobility patterns, using cluster analysis of objective measures (e.g., GPS or cellular trace data) to identify microforest features most associated with increased visitation. We will use an interrupted time series analysis to evaluate the effectiveness of the microforest intervention on lowering regional temperatures and pollution levels [60]. The interrupted time point will be defined as the date of the microforest planting. Time series data will be aggregated to weekly values pre- and post-intervention for analysis. We will fit Fourier terms in our model to capture seasonal patterns in our data [61]. In future exploratory analyses, we could also consider examining the effect of specific aspects of the greening intervention on human mobility patterns. We will use cluster analysis to identify the specific characteristics of the microforest and its environment that are most strongly associated with changes in human mobility patterns. We can use objective measures of mobility, such as GPS tracking or cellular phone traces, to determine the frequency and duration of visits to different types of green spaces. This analysis will help us to better understand the mechanisms underlying the relationship between green space and health [62,63].

#### 2.9.6. Environmental Data Analysis

Meteorological data from the Tempest and Campbell Scientific stations will be quality-controlled to identify and correct erroneous or missing values. Data will be averaged over 15 min intervals for analysis. Two primary analyses are planned: (1) Intra-park analysis—hourly park-wide temperature averages on synoptically stable days (clear skies and calm conditions) will be compared across stations to identify spatial variability due to shading, ventilation, and ground cover. Anomalies relative to park-wide averages will be correlated with solar radiation, wind speed, and ground cover type; and (2) Post-intervention analysis—post-planting data will be compared with baseline measurements to quantify immediate and long-term microclimate effects of the intervention. Long-term trends will be assessed using the central Campbell Scientific station, with monthly statistics computed for all variables. Data will be compared with other stations in the University of Louisville network and with National Weather Service stations to evaluate the microforest’s contribution to reducing the urban heat island effect. Statistical comparisons will use nonparametric tests (e.g., Wilcoxon rank-sum), with significance defined as *p* < 0.05.

Biological and Ecological Data Analysis Avian data will be analyzed in RStudio (v4.3.1) to compare species abundance and diversity across sites (Trager Microforest, University Quad, Nettleroth Sanctuary). Microsoft Excel will support visualization. Surveys will continue after planting to assess temporal changes in avian presence.

Soil flux data (CO_2_ and CH_4_) will be analyzed using multiple linear regression models, with explanatory variables, including vegetation presence, soil moisture, soil temperature, and photosynthetically active radiation (PAR). Variables showing multicollinearity (r ≥ 0.60) will not be included in the same model. Candidate models will be evaluated using Akaike’s Information Criterion corrected for small sample size (AICc), with models within ΔAICc < 2 considered equivalent.

To analyze soil microbiome data, sample inference from amplicon data will first be performed using the DADA2 denoising workflow [64]. DADA2 generates amplicon sequence variant (ASV) tables, which allow for finer resolution than older OTU grouping methods [65]. Bacterial ASVs will then be annotated using the SILVA SILVA_SSU_r138_2019 database [66], and fungal ASVs will be annotated using the UNITE sh_general_release_all_10.05.2021 database [67]. Changes in microbiome community composition over time and in response to other soil properties (e.g., soil nutrients and heavy metal concentrations) will be assessed using principal components analysis. Changes in community diversity over time will be assessed using ANOVA. Indicator species analysis will be performed to identify particular microbial species associated with increased soil health factors and greenness.

Tree inventory data will be analyzed with i-Tree Eco (v6.1.47) to estimate carbon storage and sequestration, pollution removal, oxygen production, and prevented runoff. The results will be aggregated to characterize overall ecosystem services provided by the microforest.

#### 2.9.7. Integrated Environmental, Ecological, and Human Health Analyses

Meteorological, biological, and clinical data will be integrated to examine linkages between environmental conditions, biodiversity, and human health outcomes. This combined analysis will allow evaluation of whether observed physiological responses are mediated by changes in temperature, air quality, or ecological activity at the site. Examples of this may include the reduction in temperature caused by exposure to the microforest, leading to an improvement in heart rate variability and blood pressure, as well as changes or differences in VOC exposure or cortisol levels due to greenness exposure.

### 2.10. Power Calculation

Power calculations were conducted using PASS 2023 (NCSS, LLC) to evaluate expected differences in slope of outcome measures between two walking sites (microforest vs. parking lot). The primary outcomes are blood pressure and heart rate variability (SDNN). Based on a previous walking study with a similar study design [64], we expect HRV (SDNN) values of 31.0 ± 6.7 and 22.6 ± 6.7 for the microforest walking and parking lot walking, respectively. Based on these effect sizes and using α = 0.05/2 = 0.025 to account for multiple comparisons, we expect that we will have >90% power with a sample size of *n* = 50 for HRV. If recruitment yields only *n* = 40 participants, power remains above 85% for both primary outcomes. Thus, this study is adequately powered to detect clinically meaningful differences in cardiovascular stress responses, even with modest variation in effect sizes. Subgroup analyses (e.g., by sex or pollutant exposure strata) are also expected to retain sufficient power. These estimates align with sample sizes used in previous studies employing similar protocols, including Lee et al., *n* = 43 [55], Koselka et al., *n* = 38 [51], and de Brito et al., *n* = 23 [54]. Collectively, these benchmarks provide confidence in the adequacy of the planned sample size for robust inference.

### 2.11. Maintenance and Ongoing Work

The Trager Microforest is a unique urban green space that requires intensive early maintenance to ensure establishment. An automated irrigation system was installed to support the survival of newly planted trees, shrubs, and groundcovers, with schedules adjusted according to rainfall. The site is inspected regularly by an in-house arborist and receives weekly maintenance visits from the construction contractor. Plants that die within the first years will be replaced; over the long term, natural competition for resources will be allowed to occur, resulting in some expected mortality. Tree mortality is anticipated at 8–11% during the establishment phase, increasing in later years as canopy closure leads to shading stress. Understory trees will be planted periodically to replace those lost to competition, maintaining species diversity and structural complexity.

### 2.12. Data Dissemination and Data Sharing

Data analysis will be conducted within a secure research environment. Upon study completion, datasets, analytic code, and statistical models will be made available through GitHub (3.19.3) to ensure transparency and reproducibility. Findings from the GRO Project will be disseminated through peer-reviewed publications and presentations at national and international conferences. In addition to academic dissemination, updates will be shared with stakeholders and the community. The Urban Design Studio website will continue to post blog updates on project progress and findings. Engagement efforts will also include outreach to urban planners, landscape architects, developers, and policymakers in Louisville and beyond. The Trager Microforest functions both as a public park and as an “urban laboratory,” providing a platform for future research on greening, health, and resilience. Potential future studies may extend to topics such as stormwater management, biodiversity, social cohesion, and climate adaptation.

## 3. Expected Results

The creation of the Trager Microforest as part of the Green Oasis (GRO) Project provides a structured framework to examine how targeted greening interventions influence both environmental and human health outcomes in dense urban cores. The randomized crossover design allows comparison of individual-level physiological responses when participants are exposed to two distinct urban environments: a densely vegetated microforest and a nearby impervious parking lot. By combining high-resolution environmental monitoring with continuous physiological measurements, this study is designed to determine whether modifications in microclimate are associated with measurable changes in cardiovascular and stress biomarkers. We expect that the microforest environment will exhibit measurable differences in local environmental conditions relative to the impervious comparison site. These differences may include lower ambient temperature, increased relative humidity, reduced solar radiation exposure, and potentially lower concentrations of air pollutants. Continuous meteorological monitoring and personal environmental exposure measurements collected during participant visits will allow these environmental differences to be quantified at the spatial and temporal scale of participant exposure.

The primary physiological outcomes of this study are HRV and blood pressure, both of which are well-established indicators of autonomic cardiovascular regulation and acute cardiovascular stress. Within the crossover design of the GRO study, we therefore expect that exposure to the cooler and more vegetated microforest environment may be associated with higher HRV values and modest reductions in systolic and diastolic blood pressure compared with the impervious parking lot condition. Such findings would be consistent with improved autonomic balance and reduced cardiovascular stress during exposure to the vegetated environment.

In addition to cardiovascular measurements, the protocol includes biochemical indicators of stress physiology such as salivary cortisol and urinary catecholamines and their metabolites (secondary outcomes). We expect that exposure to the microforest environment may be associated with lower peak cortisol concentrations and reduced overall cortisol responses during the exposure period compared with the impervious environment. Urinary measurements of catecholamines and their metabolites provide additional indicators of sympathetic nervous system activation. Exposure to the microforest environment may therefore be associated with lower concentrations of norepinephrine and epinephrine metabolites relative to the parking lot condition, reflecting reduced activation of the sympathetic stress response during exposure to the vegetated setting. We also expect that participants exposed to microforest environments may report lower levels of acute anxiety, reduced perceived distress, and lower rates of rumination as reflected by their scores on validated questionnaires of stress and emotional state.

Although a substantial body of literature demonstrates that exposure to green environments is associated with reduced stress, improved cardiovascular function, and increased longevity [1], evidence is limited regarding whether compact, high-density greening interventions in highly built environments can generate meaningful physiological and ecological benefits. By integrating meteorological, clinical, and ecological assessments, the GRO Project provides a multidisciplinary assessment of how microforests can alter local environments and human physiology. Integrated analysis will examine whether differences in environmental exposures explain observed changes in cardiovascular and stress biomarkers (Aim 5). For example, mediation analyses will evaluate whether reductions in ambient temperature are associated with improvements in HRV or reductions in blood pressure, or whether lower exposure to pollutants is associated with changes in cortisol or catecholamine responses. By integrating environmental, physiological, and psychological measurements within the randomized crossover framework, the GRO Project will allow examination of potential pathways linking microclimate modification to acute cardiovascular and stress responses. Furthermore, longitudinal follow-up across multiple years (2025–) will capture the maturation of the microforest, permitting evaluation of both immediate and long-term impacts.

A key strength of this project is its ability to simultaneously assess microclimate modification and human health responses. Continuous monitoring of temperature, humidity, and air quality allows direct quantification of the extent to which dense plantings mitigate heat exposure and pollutant levels at the street scale (Exploratory Aim 3). This information is critical, as climate models predict rising urban heat exposure and associated health burdens in the coming decades [48]. By linking these data to individual-level cardiovascular and stress biomarkers, the GRO Project is designed to examine whether microclimatic improvements translate into measurable physiological resilience.

The layered planting design, which mimics natural forest succession, also provides an opportunity to evaluate the ecological services of urban microforests. Baseline surveys of avian species, soil microbial activity, greenhouse gas fluxes, and tree ecosystem services (Exploratory Aim 4) establish a robust ecological benchmark against which post-intervention changes can be assessed. Integrating these ecological metrics with meteorological and health data offers novel insights into the interconnections between biodiversity, environmental function, and human health.

Equally important, this project addresses questions of scale and replicability. While large urban parks have well-documented health and climate benefits, fewer studies have examined whether small, densely planted lots, common in land-constrained downtown areas, can generate similar effects. If successful, the GRO model is designed to generate data that may inform future scalability in urban contexts with similar density and land cover characteristics, particularly in neighborhoods with limited space, high impervious-surface coverage, and elevated vulnerability to heat stress.

The policy implications of this work are substantial. The results will inform municipal decision-making related to land-use codes, tree ordinances, and urban greening priorities. By demonstrating the environmental and health benefits of compact greening interventions, this study may support more widespread adoption of evidence-based planting practices that promote resilience, reduce pollution, and improve public health. Moreover, the Trager Microforest functions as both a community amenity and a living laboratory, strengthening engagement among scientists, planners, and the public.

In summary, the GRO Project provides a comprehensive, multidisciplinary framework for evaluating how small-scale, dense greening interventions affect urban microclimates, ecological function, and cardiovascular resilience. Findings from this project are expected to advance scientific understanding, inform policy, and offer a new framework for integrating nature-based solutions into the fabric of dense urban environments. As urban populations expand and climate stressors intensify, the importance of such targeted, evidence-based interventions will only grow.

## 4. Limitations and Future Directions

Despite its strengths, the GRO Project has several limitations. First, as a single-site intervention, findings may be influenced by the unique environmental and social context of downtown Louisville, which could limit generalizability to other urban areas with different climates, vegetation types, or community structures. The Trager Microforest’s species composition and density are tailored to Louisville’s temperate climate. Replication across diverse geographic and demographic settings will be necessary to confirm the broader applicability of microforest interventions, as ecological and microclimatic outcomes may vary in other settings. Second, while the randomized crossover design strengthens causal inference, acute physiological responses may not fully capture the long-term health benefits of greening. Repeated exposure over months or years may yield different or cumulative effects that extend beyond those observed during single visits. Planned longitudinal assessments will help address this gap, but continued follow-up will be important to gauge the durability of benefits as the forest matures. Third, long-term cardiovascular disease outcomes are not measured in this study design.

Lastly, while the environmental and ecological measurements are comprehensive, they may omit key mediators such as volatile organic compounds emitted by plants, microbial aerosols, or finer indicators of biodiversity. Incorporating additional measures, such as metabolomic or microbiome analyses of participants, could further elucidate mechanisms linking greenness and health. Finally, the project is resource-intensive, requiring specialized instrumentation, personnel, and ongoing maintenance. Widespread implementation of microforests will therefore require cost–benefit analyses and evaluations of scalability across different budgetary and governance contexts.

Despite these limitations, the GRO Project establishes a foundation for future research on how small-scale greening interventions affect community-level outcomes, including social cohesion, mobility, and perceptions of safety. Integration of environmental, health, and behavioral data with geospatial modeling could inform citywide strategies for optimizing greening. By extending research beyond physiological endpoints to include social and economic dimensions, future work can help build a comprehensive evidence base to guide urban policy and promote equitable health benefits from greening initiatives.

## Figures and Tables

**Table 1:** Summary table outlining research aims, hypotheses, and outcome measures.

Aim	Hypothesis	Exposure	Outcome Measures
Primary Aim 1	Exposure to microforest improves HRV and lowers BP vs. parking lot	Microforest vs. impervious site	heart rate variability (HRV) and blood pressure (BP)
Secondary Aim 2	Exposure to microforest improves stress responses and decreases VOC exposure	Microforest vs. impervious surface	Salivary cortisol, urinary catecholamines and their metaboiltes, urinary VOC metabolites, self-reported stress and rumination.
Exploratory Aim 3	Microforest reduces heat and pollution	Pre/post intervention; spatial monitoring, comparison with adjacent impervious surface.	Temperature, humidity, PM, NO_2_, BC
Exploratory Aim 4	Microforest enhances ecological function	Pre/post intervention	Avian diversity, microbiome composition, GHG flux, ecosystem services
Integrated Aim 5	Environmental modification mediates physiological benefits	Integrated environmental metrics	HRV, BP with environmental covariates

**Table 2:** Meteorological variables recorded by Tempest and Campbell Scientific stations (temperature is measured with passively aspirated sensors in the Tempest stations and with fan-aspirated sensors in the Campbell Scientific station).

Variable	Tempest Stations	Research Station
Temperature (°C)	Passively Aspirated	Fan-Aspirated
Relative Humidity (%)	Passively Aspirated	Fan-Aspirated
Barometric Pressure (hPa)	√	√
Wind speed (m s^−1^)	√	√
Wind Direction (°)	√	√
Dewpoint Temperature (°C)	Calculated	Calculated
Solar Radiation (W m^−2^)	√	√

**Table 3:** Demographic characteristics of study participants in Wave 1 (2023) and Wave 2 (2024).

	Wave 1 (2023)	Wave 2 (2024)
Age	39 [28 to 42]	42 [29 to 52]
Sex		
Male	16 (40%)	20 (40%)
Female	24 (60%)	30 (60%)
Race		
NHW	30 (75%)	28 (56%)
NHB	3 (8%)	10 (20%)
HW	0 (0%)	3 (6%)
HB	0 (0%)	0 (0%)
Other	7 (17%)	9 (18%)
Education		
≤4-year degree	21 (53%)	27 (56%)
>4-year degree	19 (47%)	22 (44%)
Income		
<$90 K	19 (47%)	27 (54%)
>$90 K	18 (45%)	21 (42%)
Declined/Don’t know	3 (8%)	2 (4%)

Continuous data reported as median (IQR). Categorical data reported as *n* (%). NH = Non-Hispanic; H = Hispanic; W = White; B = Black.

**Table 4:** Components of the in-person exam.

Procedure	Description
Anthropometry	Weight (kilograms), height (centimeters), body mass index (BMI) calculated from weight and height measurements.
Saliva samples	Four 1 mL saliva samples collected into cryovials in 25 min intervals using the passive drool method. Salivary sample concentrations are analyzed using a cortisol curve.
Spot urine samples	Two >25 mL spot urine samples collected in sterile urine cups at arrival and 30 min post-exposure. Stored for analysis of VOC metabolites, nicotine, and catecholamine metabolites.
Axillary temperature *	Axillary (or armpit) temperature recorded at 12 s intervals continuously throughout the duration of the outdoor testing period.
Blood pressure +	Brachial blood pressure collected via an ambulatory blood pressure cuff set to measure at 5 min intervals throughout the duration of the outdoor testing period.
Electrocardiogram	Electrocardiogram (ECG) designed for remote monitoring applications recorded ECG continuously for the duration of the outdoor testing period. ECG data stored in multiple formats for heart rate variability (HRV) analysis in third party software (i.e., Kubios HRV).

* Not implemented in Wave 1. + Different blood pressure devices used in Wave 1 versus Waves 2 and 3.

## Data Availability

The original data presented in the study are openly available in Box at https://louisville.app.box.com/folder/352361324550?s=29f8ytcwz8u7czsc07kmy6bkhdaaebbu (accessed on 28 January 2026). Future data collected for the TMFP will be available at the same URL. Clinical data are not available due to patient privacy.

## Supplementary Materials

The following supporting information can be downloaded at: https://www.mdpi.com/article/10.3390/ijerph23030365/s1, Table S1: Components of the electronic questionnaire; Table S2: Species list of canopy and understory trees planted in the Trager Microforest. Includes common and scientific names, families, and functional groupings (deciduous vs. evergreen); Table S3: Species list of shrubs planted in the Trager Microforest, with common/scientific names, growth form, and placement within the planting design; Figure S1: Schematic of the post-construction Trager Microforest, indicating locations of meteorological sensors; Figure S2: Installation of rear metal signage and fins; Figure S3: Photographs of the Trager Microforest before and after planting (top row) and progression of construction (bottom row). Right-most photo on the top row is courtesy of Gresham Smith.
