# Supplementary material for: Evaluating Microclimate Modification and Acute Cardiovascular Stress Responses to a Dense Urban Microforest: The Green Oasis (GRO) Protocol
Source: Int J Environ Res Public Health. 2026 Mar 13;23(3):365. doi: 10.3390/ijerph23030365 (PMC13026838; doi:10.3390/ijerph23030365)

TABLE S1. Components of the electronic questionnaire.

Survey Information Collected	Description
Administered before In-Person Visit	
Personal information	Name, date-of-birth, biologic sex (male, female, or intersex), race (Black, White, other [American Indian/Alaskan Native, Asian, Hawaiian/Pacific Islander, other], ethnicity (Hispanic or Non-Hispanic), age, education (high school or below, some college, 2- year college degree, 4-year college degree, graduate-level degree), annual household income (from <$20,000 to >$125,000), and occupational type and status
Contact information	Address, email, and telephone
Informed consent	Obtained signed informed consent that complies with all required standards
Permission to contact for any future studies	Yes or no
Women's Health	Only applicable for sex selected "female". Pregnancy status, menstrual frequency, hysterectomy, menopause or birth control hormone medications.
Perceived Stress Scale (PSS-10)	The questionnaire assesses the degree to which current (within the last month) life situations are perceived as stressful. Measures perceived helplessness and lack of self-efficacy.
Five Facet Mindfulness Questionnaire: Short-Form (FFMQ-SF)	The questionnaire assesses five aspects of mindfulness. The facets include: Observing, describing, acting with awareness, non-judgement, and non-reactivity.
Multidimensional Assessment of Interoceptive Awareness (version 2) (MAIA-2)	The questionnaire assesses interoception (or awareness of bodily sensations) across 8 scales that represent 5 dimensions of bodily awareness. The scales include: Noticing, not-distracting, not-worrying, attention regulation, emotional awareness, self-regulation, body listening, and trust.
Nature Survey (including the Extended Inclusion of Nature in Self scale (EINS))	The questionnaire assesses time spent outdoors, time spent in nature, and interconnectedness with nature.
Family History of Cardiovascular Disease	Heart attack (or myocardial infarction) status and count of full-blooded relatives.
Allergies	Allergy status, treatment method, and impact on daily living.

Behavioral Habits	Alcohol and marijuana consumption patterns and physical activity habits.
Tobacco Habits	Tobacco type, use patterns, history, and secondhand exposure risks.
Medication use	Current use of medications for cardiovascular risk factor reduction and cardiovascular disease.
**Survey Information Collected**	**Description**
Administered during In-Person Visit	
Subjective Units of Distress Scale (SUDS)	The questionnaire assesses the current level of distress on a single scale from 0-100.
State-Trait Anxiety Inventory (STAI)	The questionnaire assesses the current “state anxiety” (or temporary anxiety) and “trait anxiety” (or more general anxiety) level through 20 Likert-scale questions.
*Brief State Rumination Inventory (BSRI)	The questionnaire uses 8 items to assess maladaptive state rumination.
*Not implemented during wave 1.	

TABLE S2. Species list of canopy and understory trees planted in the Trager Microforest. Includes common and scientific names, families, and functional groupings (deciduous vs. evergreen).

**Common Name**	**Scientific Name**	**Min Size**	**Family**	**Quantity**	**Type**	**Functional Group**	**Code**
Dura Heat River Birch	Betula nigra \`BNMTF\`	10' ht	Betulaceae	1	Canopy	Deciduous	BENB
Kentucky Coffeetree	Gymnocladus dioicus \`Espresso\`	3" cal	Fabaceae	2	Canopy	Deciduous	GYDE
Skinny Lattee Kentucky Coffee Tree	Gymnocladus dioicus 'Morton'	3" cal	Fabaceae	4	Canopy	Deciduous	GYDS
Slender Silhoutte Sweet Gum	Liquidambar styraciflua \`Slender Silhouette\`	3" cal	Altingiaceae	3	Canopy	Deciduous	LSSS
Prystal Tulip Poplar	Liriodendron tulipifera \`LTMTF\`	3" cal	Magnoliaceae	2	Canopy	Deciduous	LITL
White Pine	Pinus strobus	8' ht	Pinaceae	4	Canopy	Evergreen	PIST
Exclamation! London Plane Tree	Platanus x acerifolia 'Morton Circle'	3" cal	Platanaceae	2	Canopy	Deciduous	PLEX

Sun Breaker Swamp White Oak	Quercus bicolor `Green Nova`	3" cal	Fagaceae	4	Canopy	Deciduous	QBGN
Shingle Oak	Quercus imbricaria	3" cal	Fagaceae	1	Canopy	Deciduous	QUIM
American Elm	Ulmus americana	5" cal	Ulmaceae	2	Canopy	Deciduous	ALUM
Lacebark Elm	Ulmus parvifolia	2.5" cal	Ulmaceae	3	Canopy	Deciduous	ULPA
Perkins Pink Yellowwood	Cladrastis kentukea `Perkins Pink`	3" cal	Fabaceae	4	Codominant	Deciduous	CKPP
Canaertii Eastern Redcedar	Juniperus virginiana `Canaertii`	6' ht	Cupressaceae	4	Codominant	Evergreen	JUVC
Firestarter Tupelo	Nyssa sylvatica `JFS-red`	3" cal	Nyssaceae	5	Codominant	Deciduous	NYSF
Norway Spruce	Picea abies	8' ht	Pinaceae	3	Codominant	Evergreen	PIAB
Pyramidal White Pine	Pinus strobus 'Fastigiata'	6' ht	Pinaceae	4	Codominant	Evergreen	PISF
Loblolly Pine	Pinus taeda	10' ht	Pinaceae	5	Codominant	Evergreen	PITA
Eastern Hemlock	Tsuga canadensis	8' ht	Pinaceae	4	Codominant	Evergreen	TSCA
Weeping Eastern Hemlock	Tsuga canadensis 'Pendula'		Pinaceae	3	Codominant	Evergreen	TSPE
Cumulus Alleheny Serviceberry	Amelanchier laevis `Cumulus`	3" cal	Rosaceae	8	Understory	Deciduous	AMLC
Fire King American Hornbeam	Carpinus caroliniana `J.N. Select A`	3" cal	Betulaceae	5	Understory	Deciduous	CCFK
Merlot Eastern Redbud	Cercis canadensis `Merlot`	3" cal	Fabaceae	3	Understory	Deciduous	CECM
Alley Cat Eastern Redbud	Cercis canadensis 'Alley Cat'	3" cal	Fabaceae	3	Understory	Deciduous	CEAC
The Rising Sun Eastern Redbud	Cercis canadensis 'JN2'	3" cal	Fabaceae	3	Understory	Deciduous	CERS
Carolina Sweetheart Eastern Redbud	Cercis canadensis 'NCCC1'	3" cal	Fabaceae	3	Understory	Deciduous	CECS
Nellie R. Stevens Holly	Ilex x 'Nellie R. Stevens'	8' ht	Aquifoliaceae	3	Understory	Evergreen	ILNE

Green Shadow Sweetbay Magnolia	Magnolia virginiana \`Green Shadow\`	3'' cal	Magnoliaceae	7	Understory	Evergreen	MVGS
Sherman Eddy Pitch Pine	Pinus rigida \`Sherman Eddy\`	2'' cal	Pinaceae	3	Understory	Evergreen	OSVG
Autumn Treasure American Hophornbeam	Ostrya virginiana 'JFS-KW5'	8' ht	Betulaceae	3	Understory	Deciduous	PISE
Cutleaf Staghorm Sumac	Rhus typhina \`Lacinata\`	4' ht	Anacardiaceae	10	Understory	Deciduous	RHTY

TABLE S**3.** Species list of shrubs planted in the Trager Microforest, with common/scientific names, growth form, and placement within the planting design.

**Common Name**	**Scientific Name**	**Container**	**QTY**	**Type**	**Code**
Bottlebrush Buckeye	Aesculus parviflora	3 gal	14	Shrub	AP
Goatsbeard	Aruncus dioicus	3 gal	10	Shrub	ARD
Dwarf Fothergilla	Fothergilla gardenii	Cont.	40	Shrub	FG
Common Witch Hazel	Hamamelis virginiana	Cont.	7	Shrub	HV
Winter Gold Winterberry	Ilex verticillata 'Winter Gold'	5 gal	12	Shrub	ILV
Little Henry Sweetspire	Itea virginica \`Little Henry\`	Cont.	34	Shrub	IV
Hughes Creeping Juniper	Juniperus horizontalis 'Hughes'	5 gal	16	Shrub	JH
Gro-Low Fragrant Sumac	Rhus aromatica 'Gro-Low'	Cont.	63	Shrub	RG
Dwarf Japanese Yew	Taxus cuspidata 'Nana'	5 gal	30	Shrub	TXN
Mohawk Viburnum	Viburnum x burkwoodii 'Mohawk'	Cont.	6	Shrub	VV
False Goatsbeard	Astilbe biternata	1 gal	29	Herbacious Perennial	ASBI
Ostrich Fern	Matteuccia pennsylvanica	Cont.	45	Herbacious Perennial	MAP
Woodland Phlox	Phlox divaricata	1 gal	73	Herbacious Perennial	PW
Obedient Plant	Physostegia virginiana	1 gal	82	Herbacious Perennial	PV
Feather Reed Grass	Calamagrostis arundinacea	1 gal	50	Ornamental Grass	CALA
Northern Sea Oats	Chasmanthium latifolium	Cont.	38	Ornamental Grass	CL

Chocolate Chip Carpet Bugle	Ajuga reptans 'Chocolate Chip'	Cont.	113	Ground Cover	AJU
Pennsylvania Sedge	Carex pensylvanica	Cont.	485	Ground Cover	CP
Big Blue Lilyturf	Liriope muscari 'Big Blue'	Cont.	113	Ground Cover	LIR
Allegheny Spurge	Pachysandra procumbens	Cont.	491	Ground Cover	PP

Figure S1. Schematic of the post-construction Trager Microforest, indicating locations of meteorological sensors.

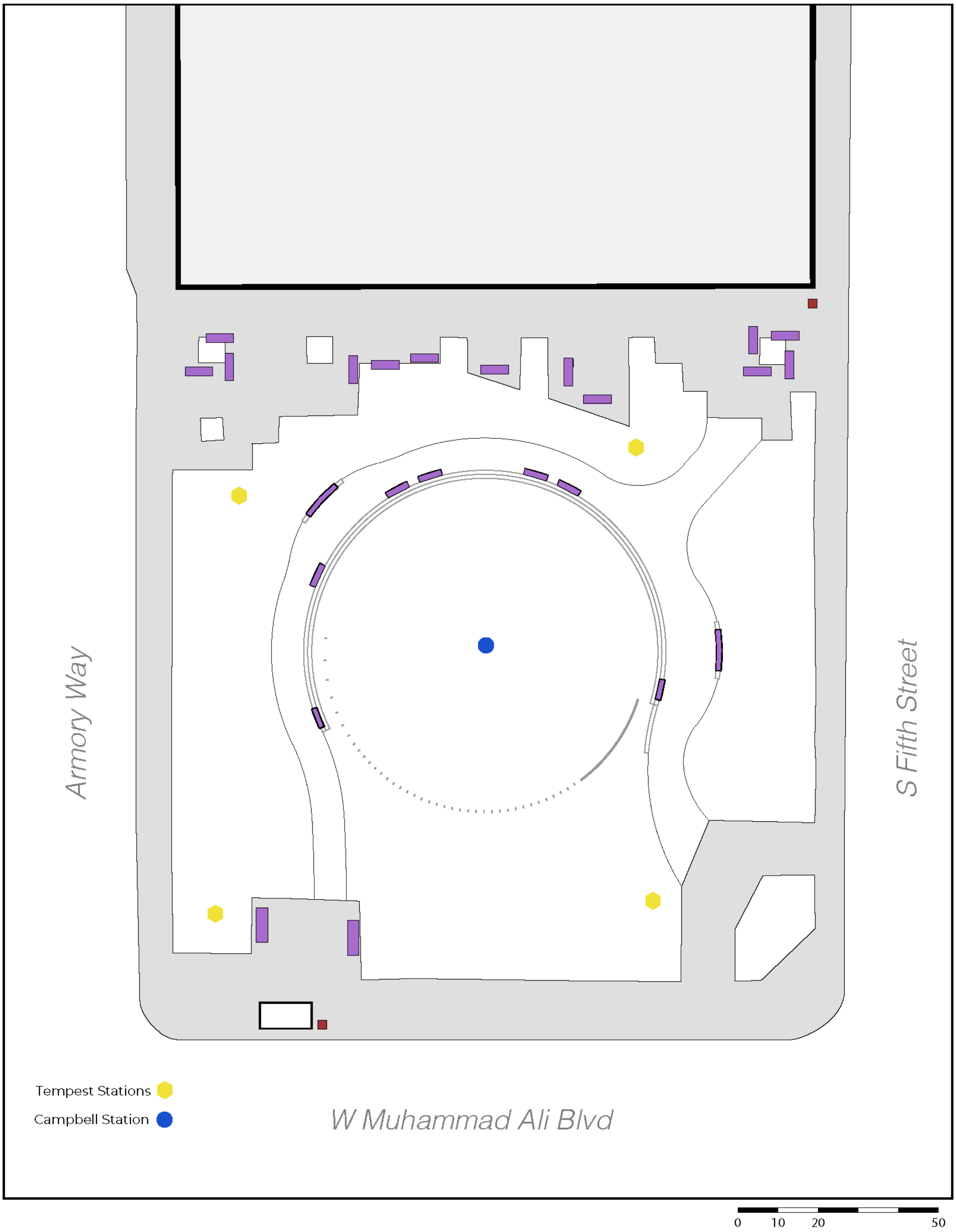

Figure S2. Installation of rear metal signage and fins.

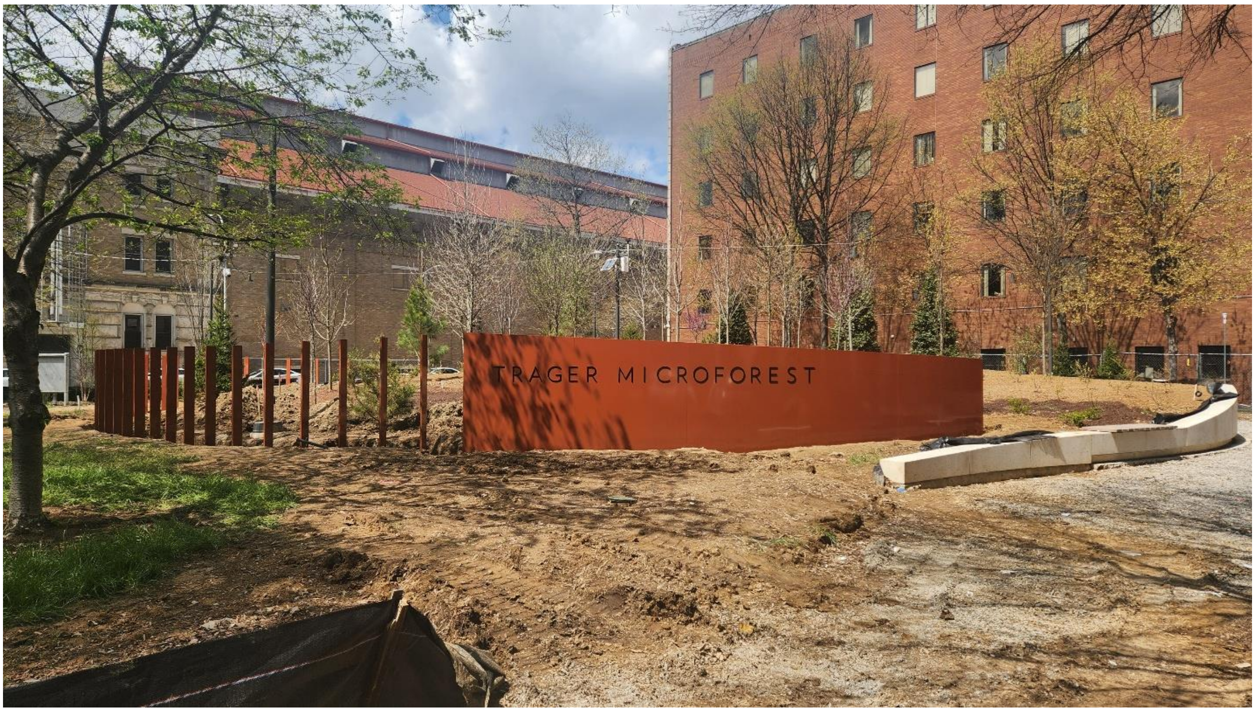

Figure S3. Photographs of the Trager Microforest before and after planting (top row) and progression of construction (bottom row).

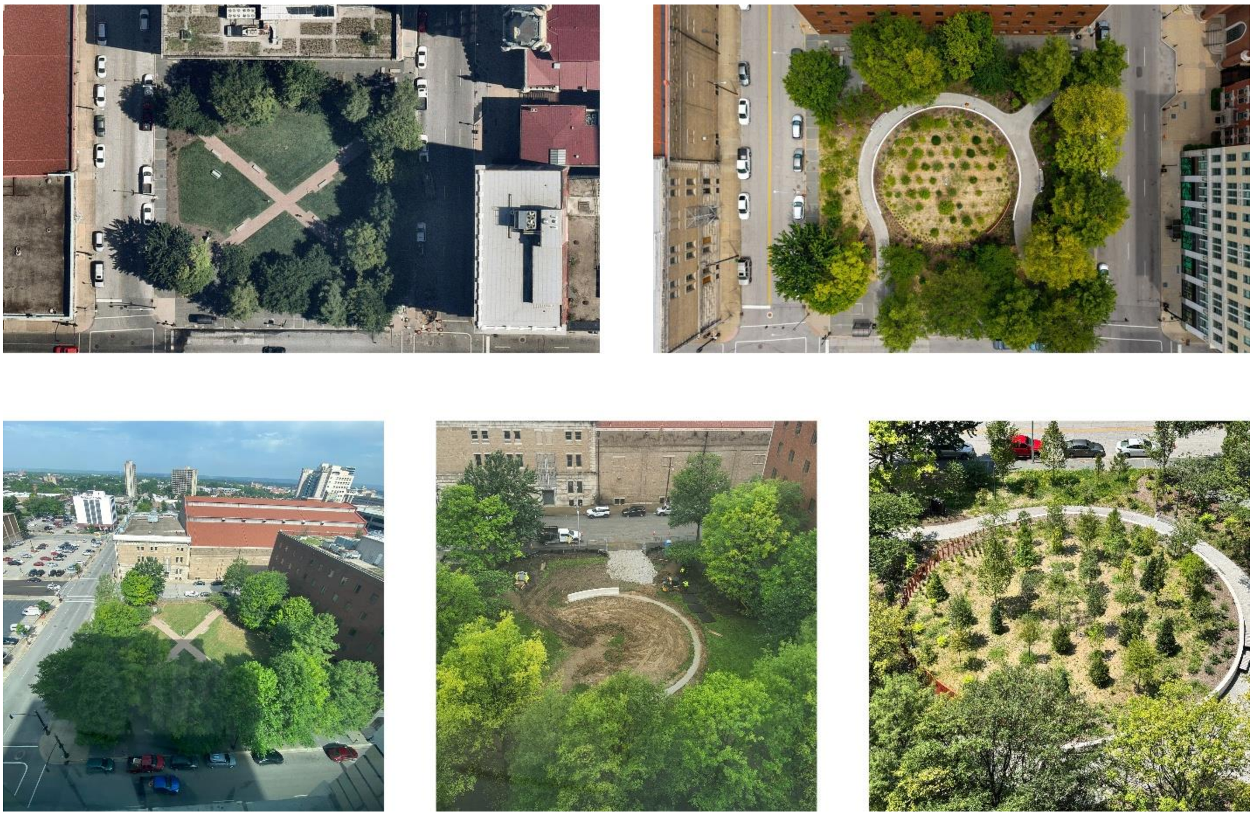

Supplement: Supplementary file 1 [file ijerph-23-00365-s001.zip › ijerph-4146980-supplementary.pdf]
